# Supplementary material for: Structural basis of agonist specificity of α1A-adrenergic receptor
Source: Nat Commun. 2023 Aug 10;14:4819. doi: 10.1038/s41467-023-40524-2 (PMC10415349; doi:10.1038/s41467-023-40524-2)
Supplement: Supplementary file 1 — Supplementary Information [file 41467_2023_40524_MOESM1_ESM.pdf]

A61603–α_{1A}-AR–Gq cryo-EM data processing

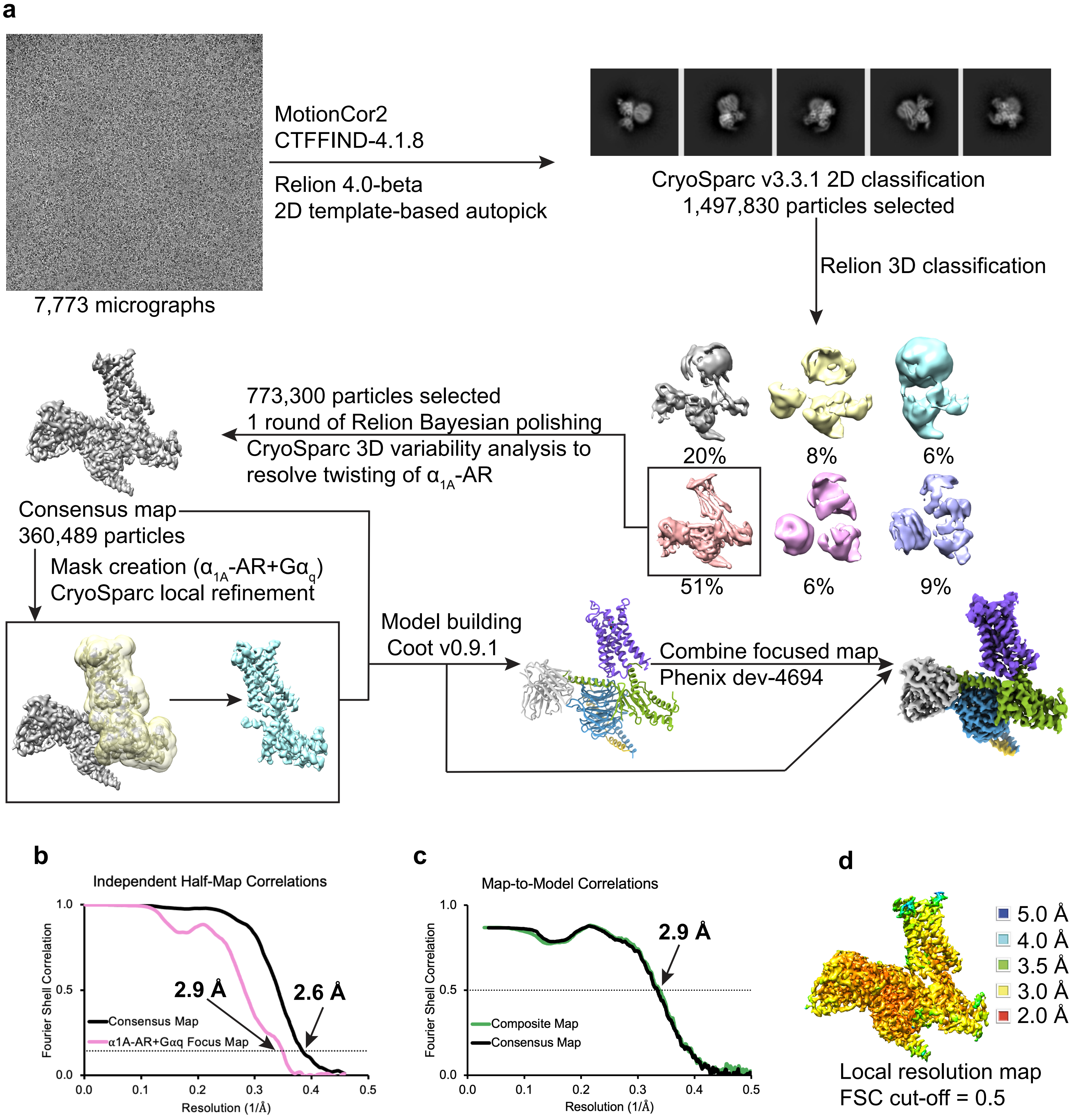

Supplementary Figure 1. A61603–α_{1A}-AR–Gq cryo-EM data processing.
a, Cryo-EM data processing workflow of A61603–α_{1A}-AR–Gq complex. **b**, Fourier shell correction curves of consensus map and α_{1A}-AR and $G\alpha_q$ focus map. **c**, Cross-validation of consensus and composite maps to A61603–α_{1A}-AR–Gq complex model. **d**, Local resolution of consensus map.

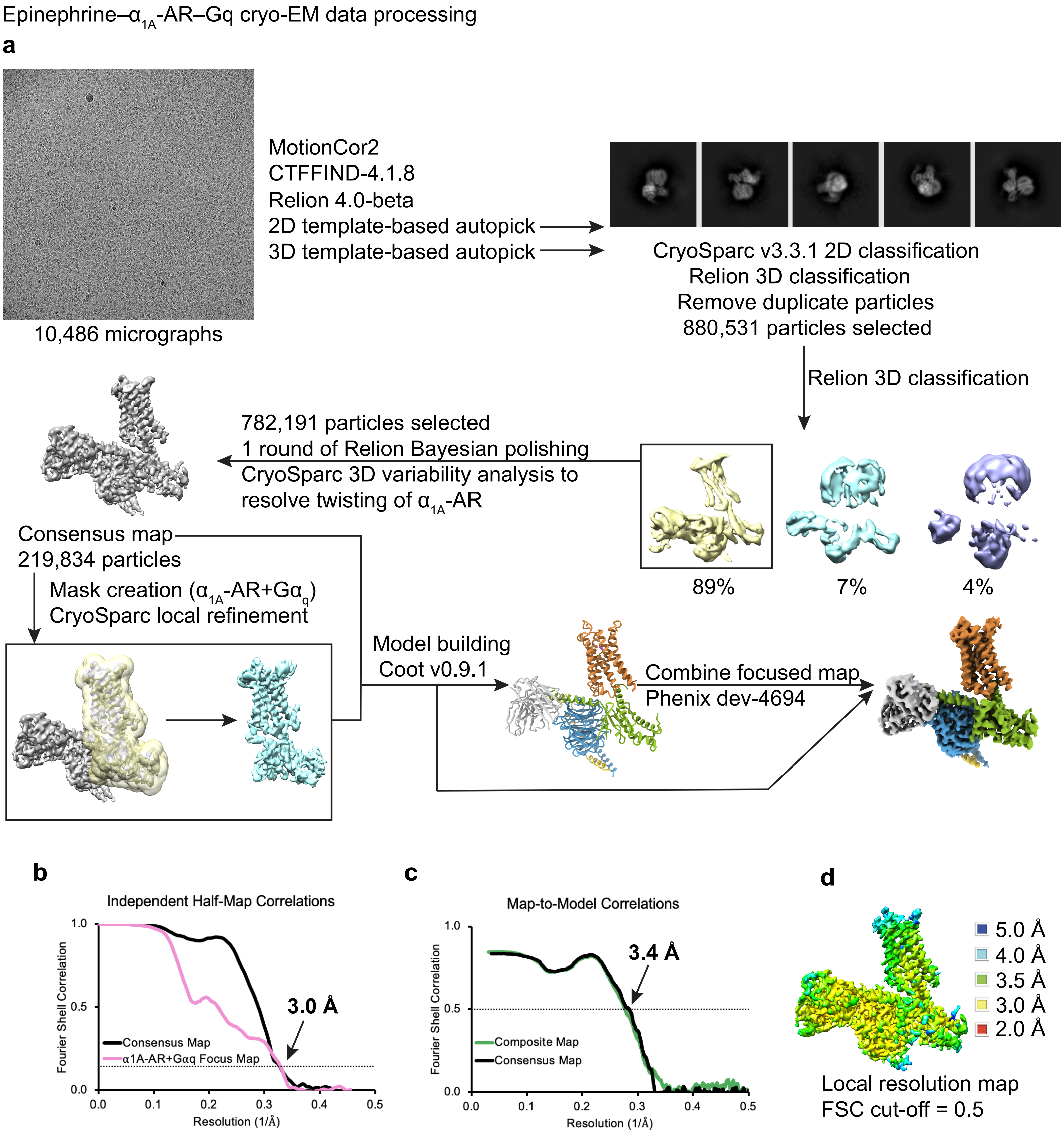

Supplementary Figure 2. Epinephrine–α$_{1A}$-AR–Gq cryo-EM data processing.
a, Cryo-EM data processing workflow of epinephrine–α$_{1A}$-AR–Gq complex. **b**, Fourier shell correction curves of consensus map and α$_{1A}$-AR and Gα$_q$ focus map. **c**, Cross-validation of consensus and composite maps to epinephrine–α$_{1A}$-AR–Gq complex model. **d**, Local resolution of consensus map.

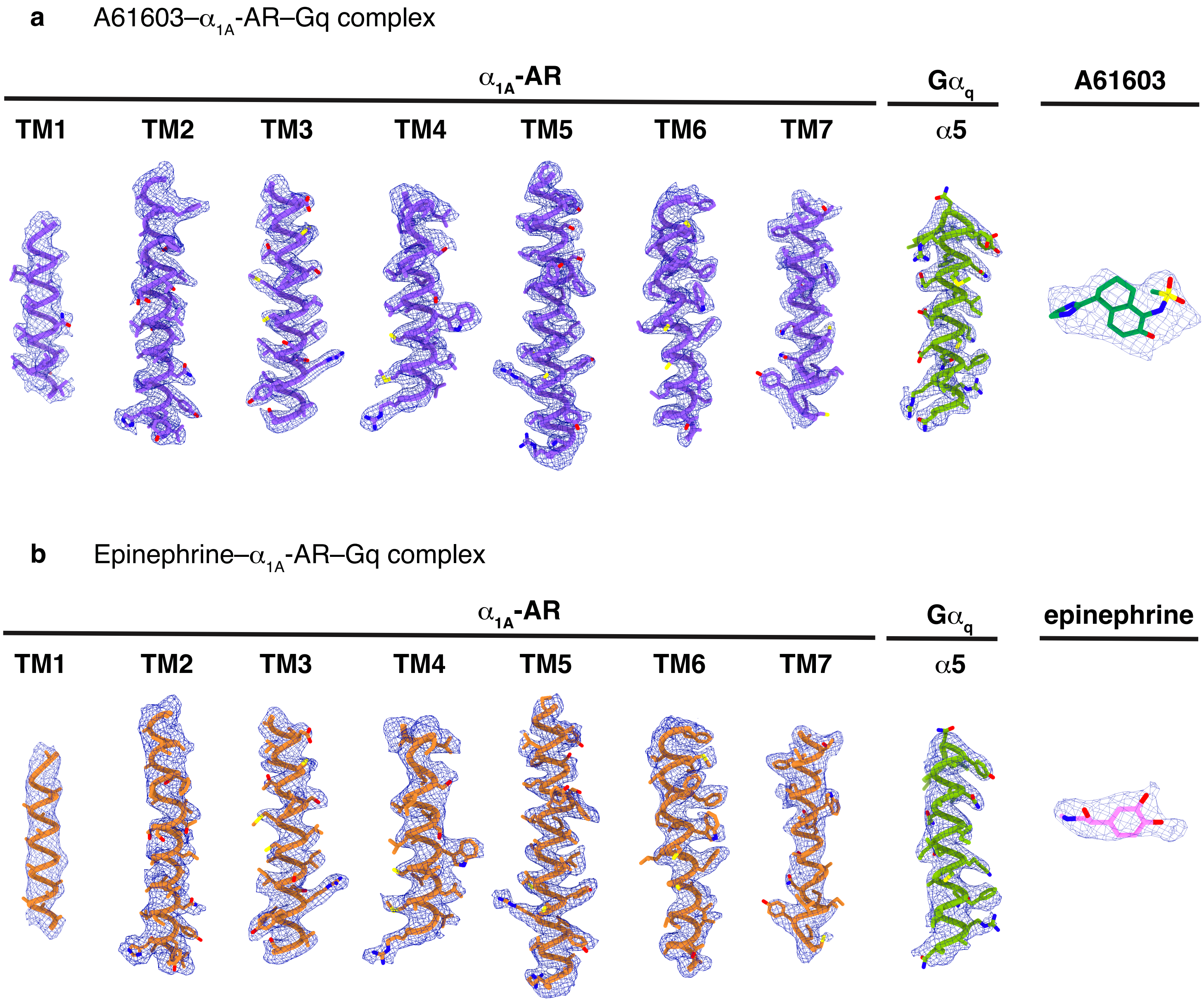

Supplementary Figure 3. Cryo-EM maps versus refined structures.
Cryo-EM density maps and models are shown for all seven transmembrane α-helices of α_{1A}-AR and α5-helix of $G\alpha_q$, of A61603–α_{1A}-AR–G_q complex **(a)** and epinephrine–α_{1A}-AR–G_q complex **(b)**.

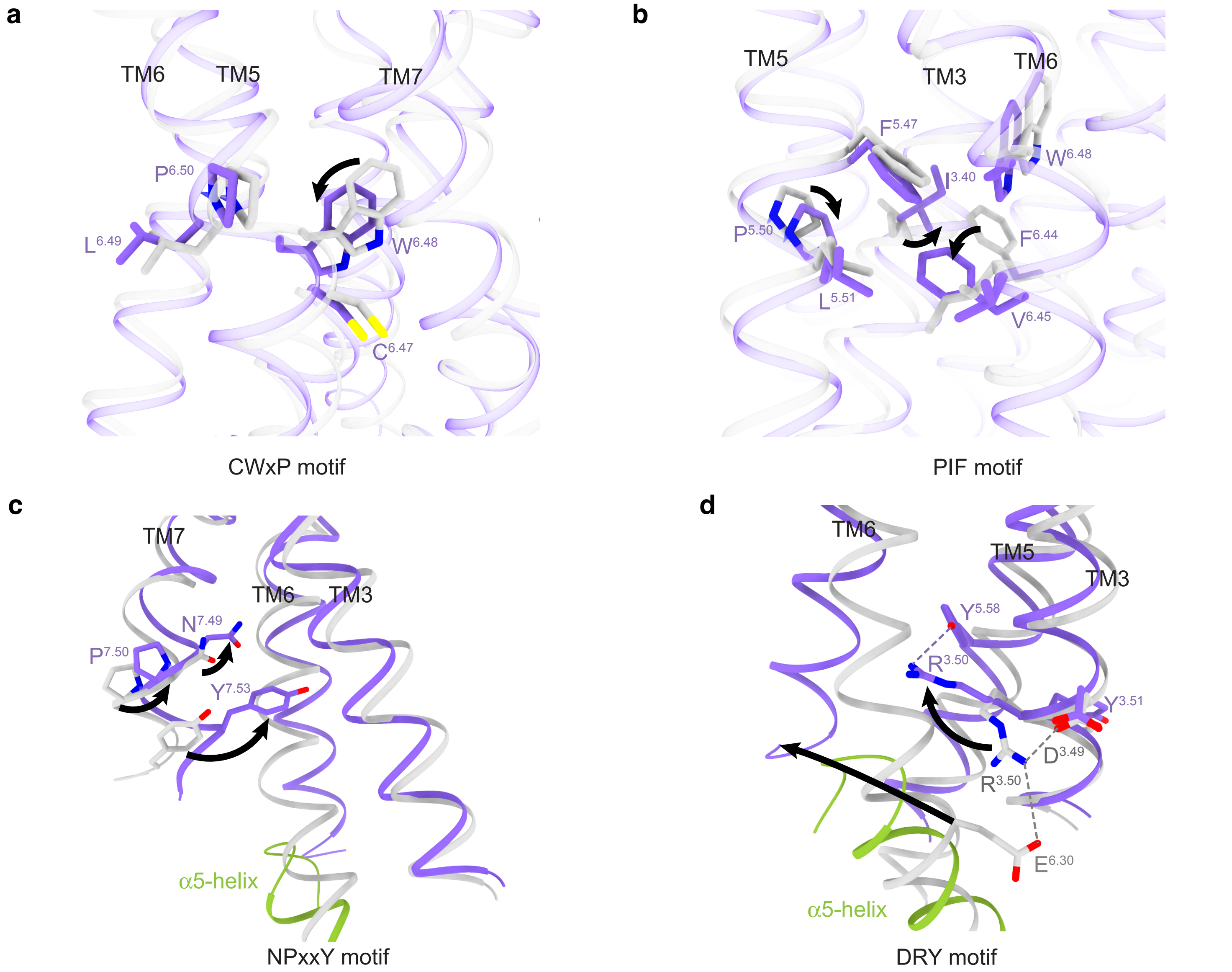

Supplementary Figure 4. Conformational changes of α_1-AR during the activation.
Structural comparisons of the inactive state α_{1B}-AR (grey; PDB 7B6W) and the active state of α_{1A}-AR in complex with Gq (this work) are shown. (**a**) The conformational changes of the CWxP motif during α_1-AR activation are shown. (**b**) The conformational changes of the PIF motif during α_1-AR activation are shown. (**c**) Movement of $Y^{7.53}$ within the NPxxY motif. (**d**) The conformational changes in the DRY motif.

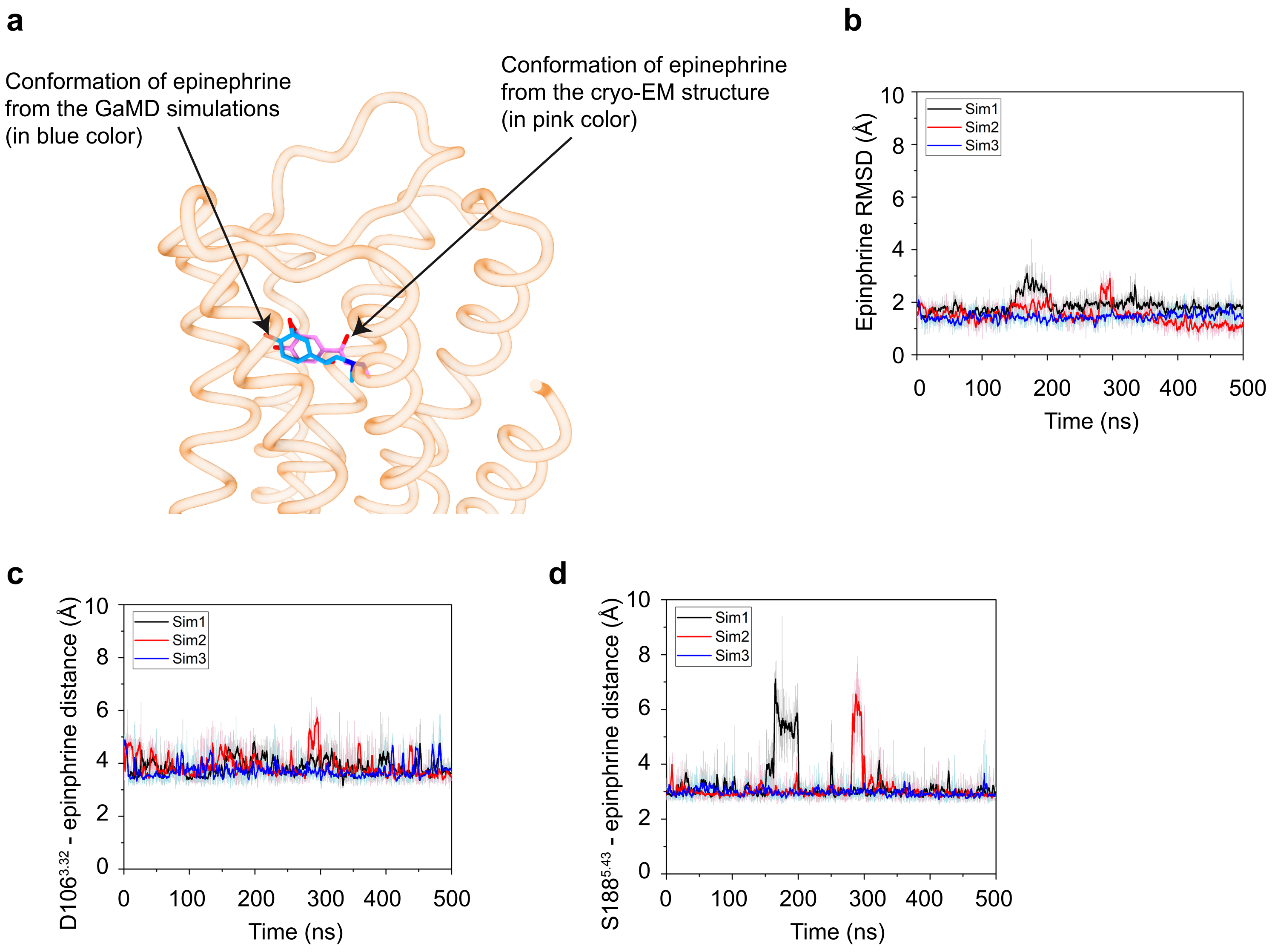

Supplementary Figure 5. **GaMD simulations of epinephrine.**
(**a**) Comparison of the conformations of epinephrine in the cryo-EM structure (in pink color) and from GaMD simulations (in blue color). (**b**) The epinephrine RMSD by comparing the conformations of epinephrine in the cryo-EM structure and from GaMD simulations. (**c,d**) time courses of the distances between epinephrine and specific residues of α_{1A}-AR were calculated from the GaMD simulations: between the CG atom of D106$^{3.32}$ and N1 atom of epinephrine (**c**), and between the OG atom of S188$^{5.43}$ and O2 atom of epinephrine (**d**). Three independent 500 ns GaMD simulations are shown for each condition.

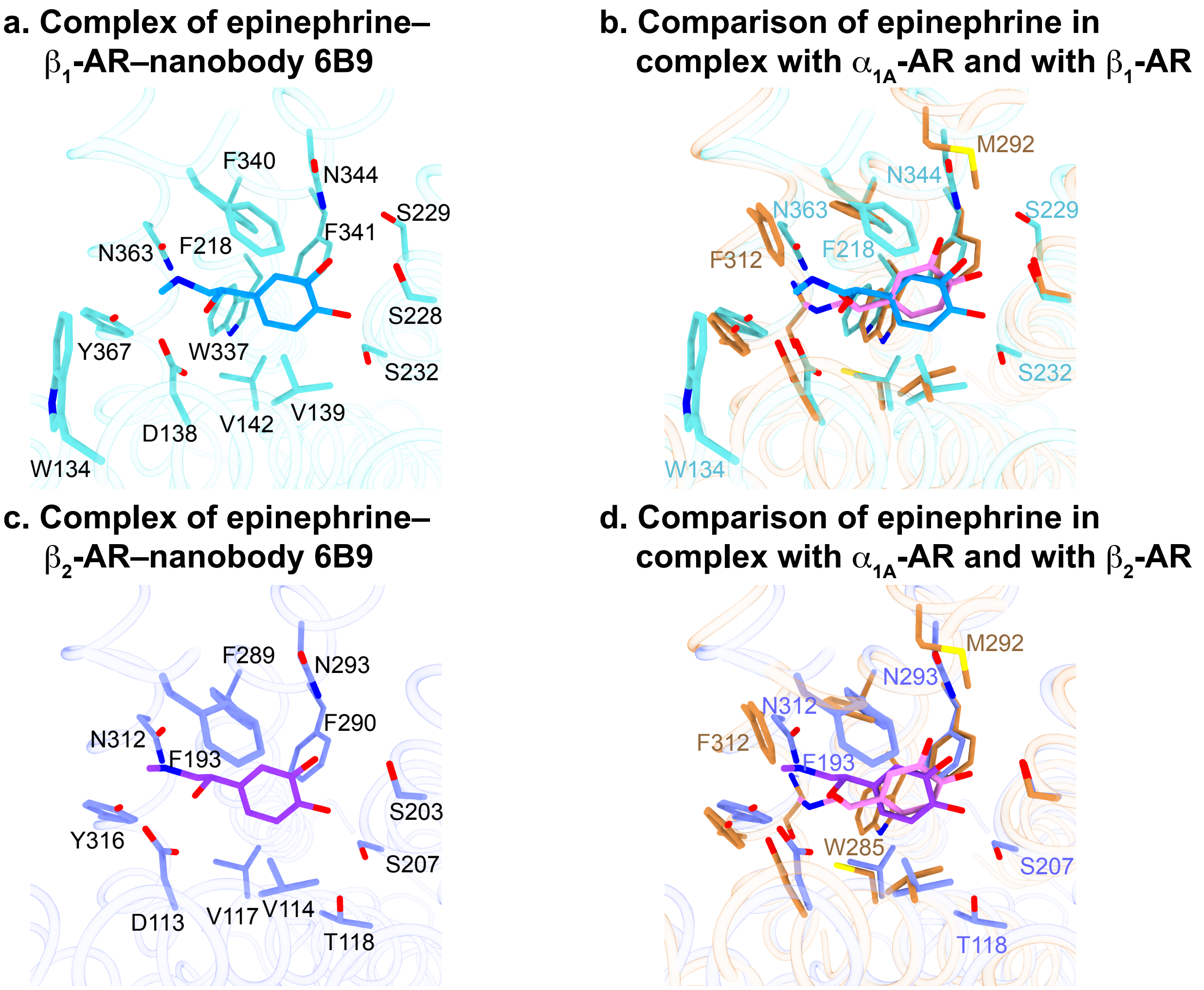

Supplementary Figure 6. **Different conformations of epinephrine.**
(a) epinephrine in the complex of epinephrine–β_1-AR–nanobody 6B9. **(b)** Comparison of epinephrine in complex with α_{1A}-AR and with β_1-AR. (**c**) Epinephrine in the complex of epinephrine–β_2-AR–nanobody 6B9. (**d**) Comparison of epinephrine in complex with α_{1A}-AR and with β_2-AR.

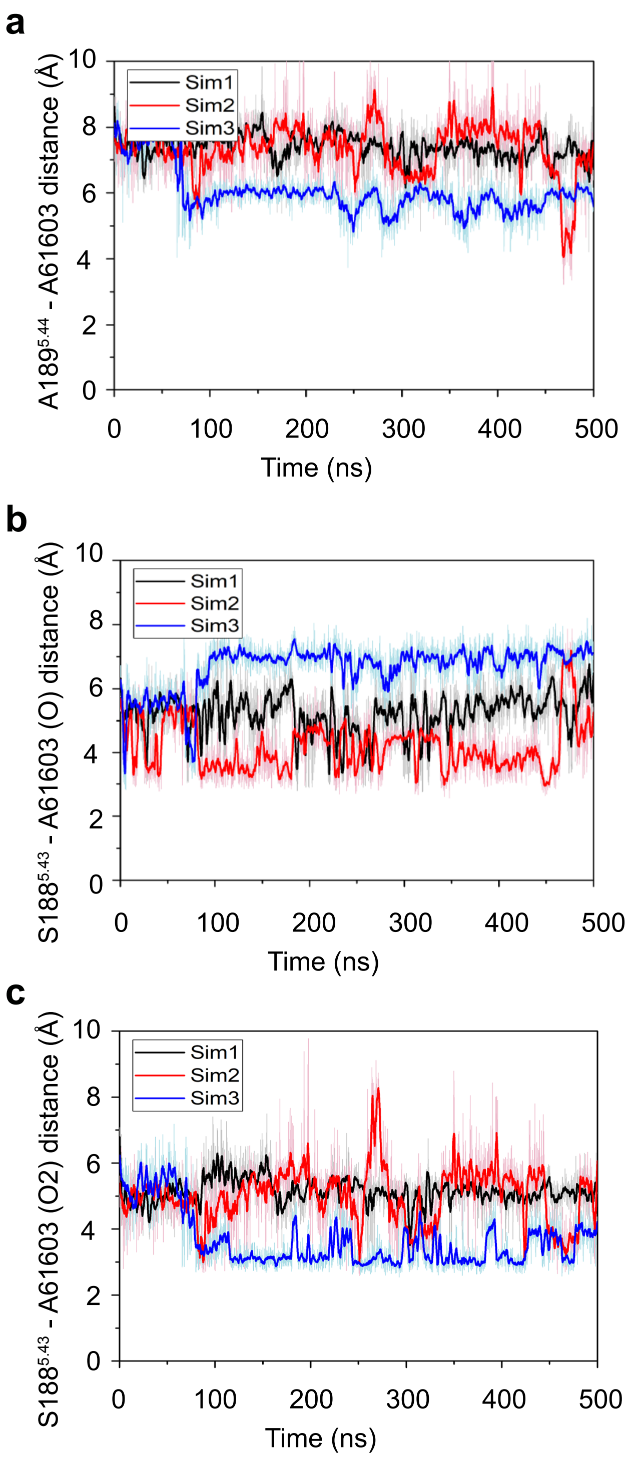

Supplementary Figure 7. **GaMD simulations of A61603.**
Time courses of the distances between A61603 and specific residues of α_{1A}-AR were calculated from the GaMD simulations: the N atom of A189$^{5.44}$ and the O2 atom of A61603 (**a**), the OG atom of S188$^{5.43}$ and the O atom of A61603 (**b**), and the OG atom of S188$^{5.43}$ and the O2 atom of A61603 (**c**). Three independent 500 ns GaMD simulations are shown for each condition.

a. Phylogenetic tree of all human ARs

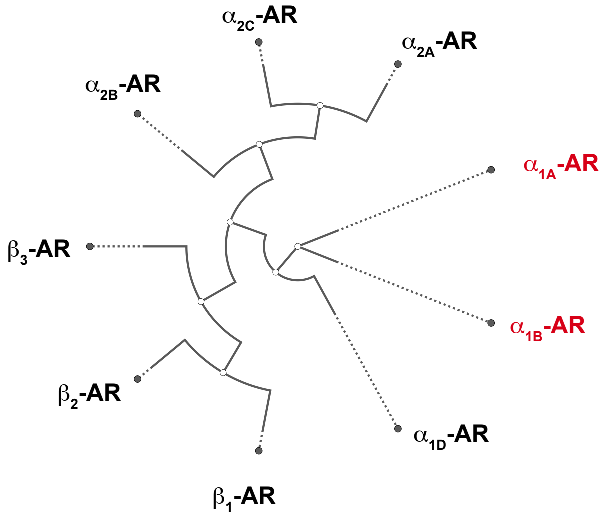

b. Most of A61603 interacting residues are conserved in α_1-ARs

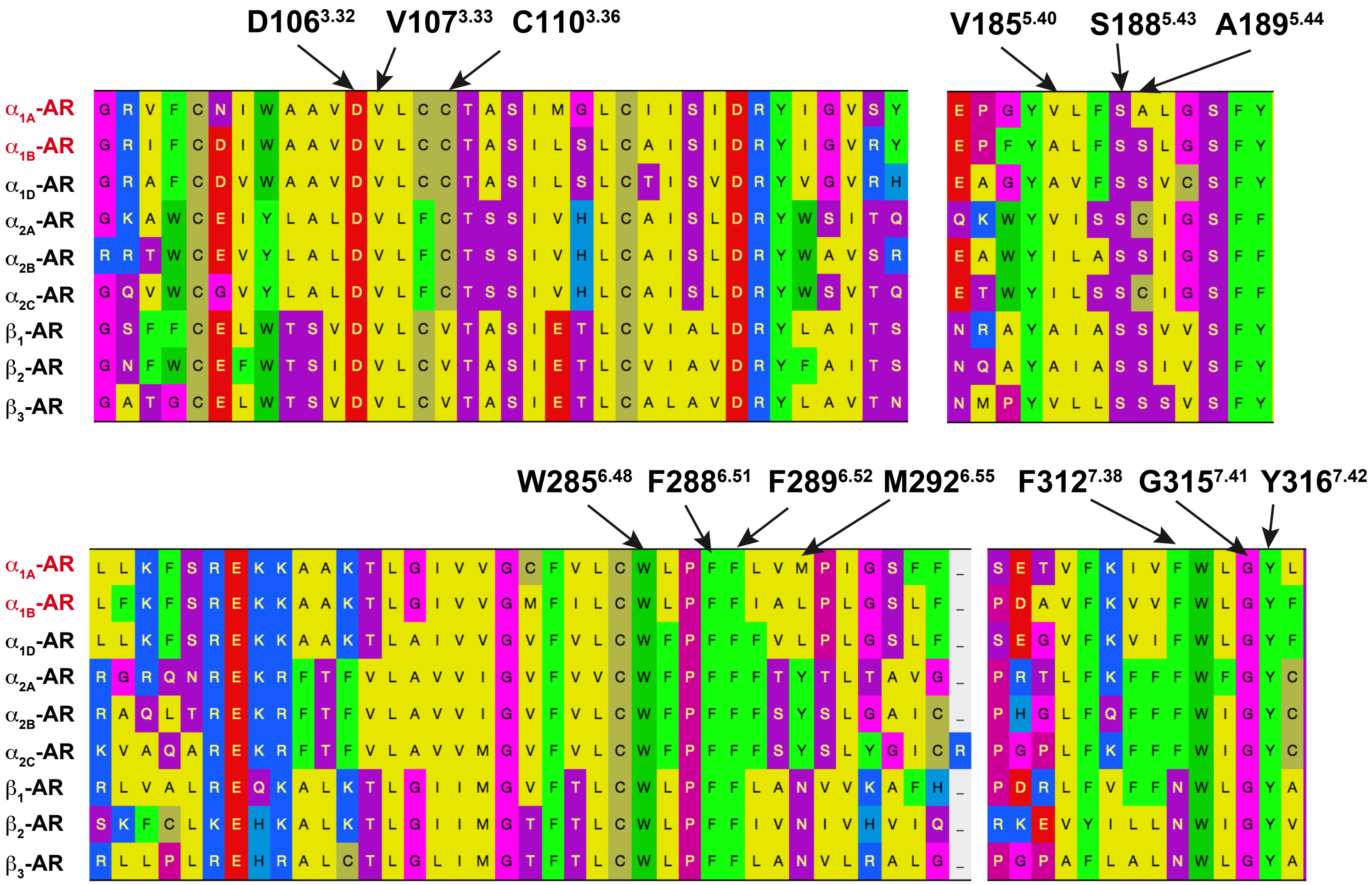

Supplementary Figure 8. Conservation assessments of all human ARs.
(a) Phylogenetic tree of all human ARs. **(b)** Structure-based sequence alignment of all human ARs generated in GPCRdb. A61603 interacting residues are labeled. As the sequence alignment indicated, most of A61603 interacting residues are conserved in α_1-ARs.

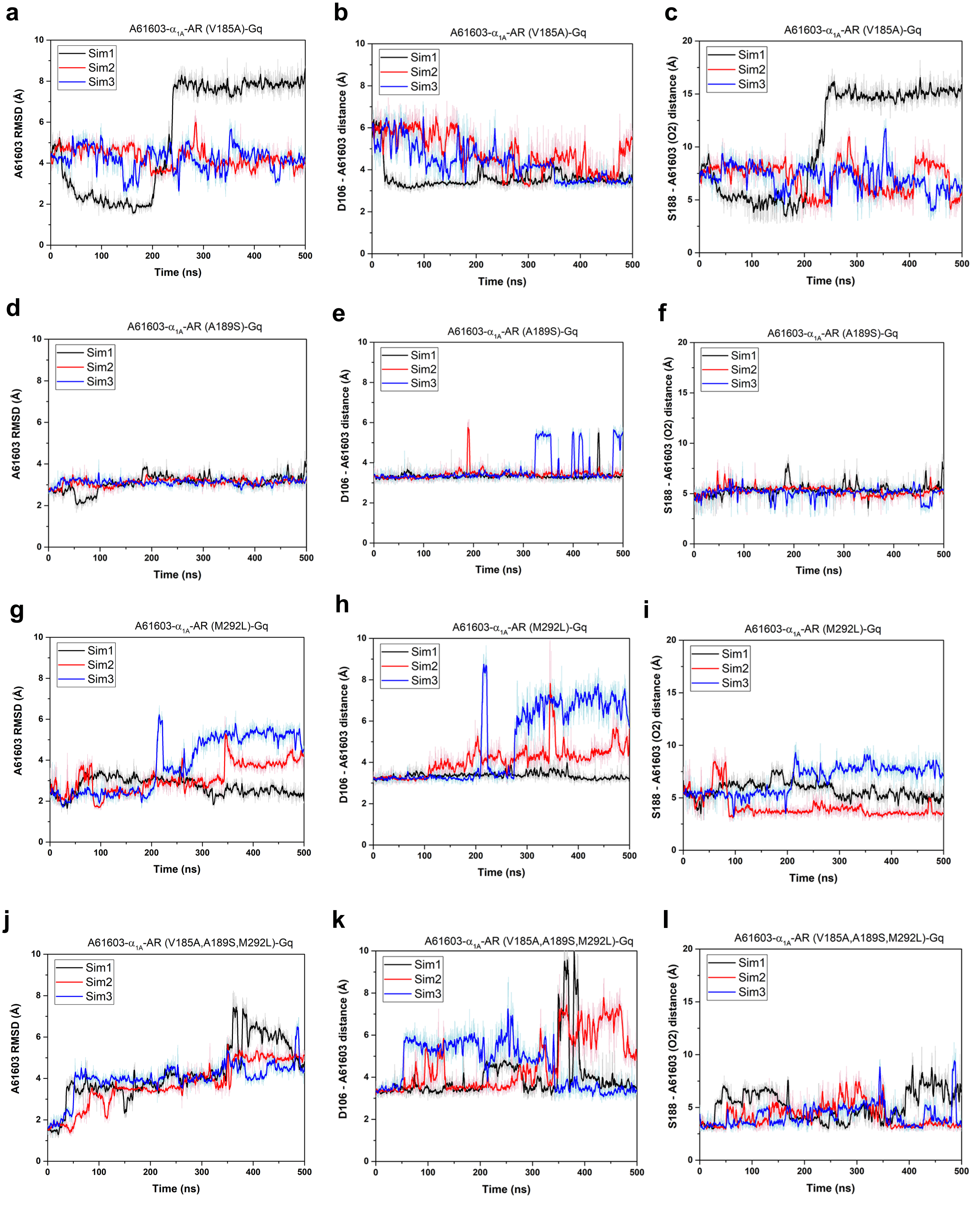

Supplementary Figure 9. **GaMD simulations of A61603 in complexes with mutant α_{1A}-ARs.** Time courses of A61603 RMSDs relative to the initial structures in the systems of α_{1A}-AR(V185A) (**a-c**), α_{1A}-AR(A189S) (**d-f**), α_{1A}-AR(M292L) (**g-i**), and α_{1A}-AR(V185A,A189S,M292L) (**j-l**). Time courses of the distances between A61603 and specific residues of mutant α_{1A}-ARs were calculated from the GaMD simulations: the CG atom of D106$^{3.32}$ and the N2 atom of A61603 (**b,e,h,k**), and the OG atom of S188$^{5.43}$ and the O2 atom of A61603 (**c,f,i,l**). Three independent 500 ns GaMD simulations are shown for each condition.

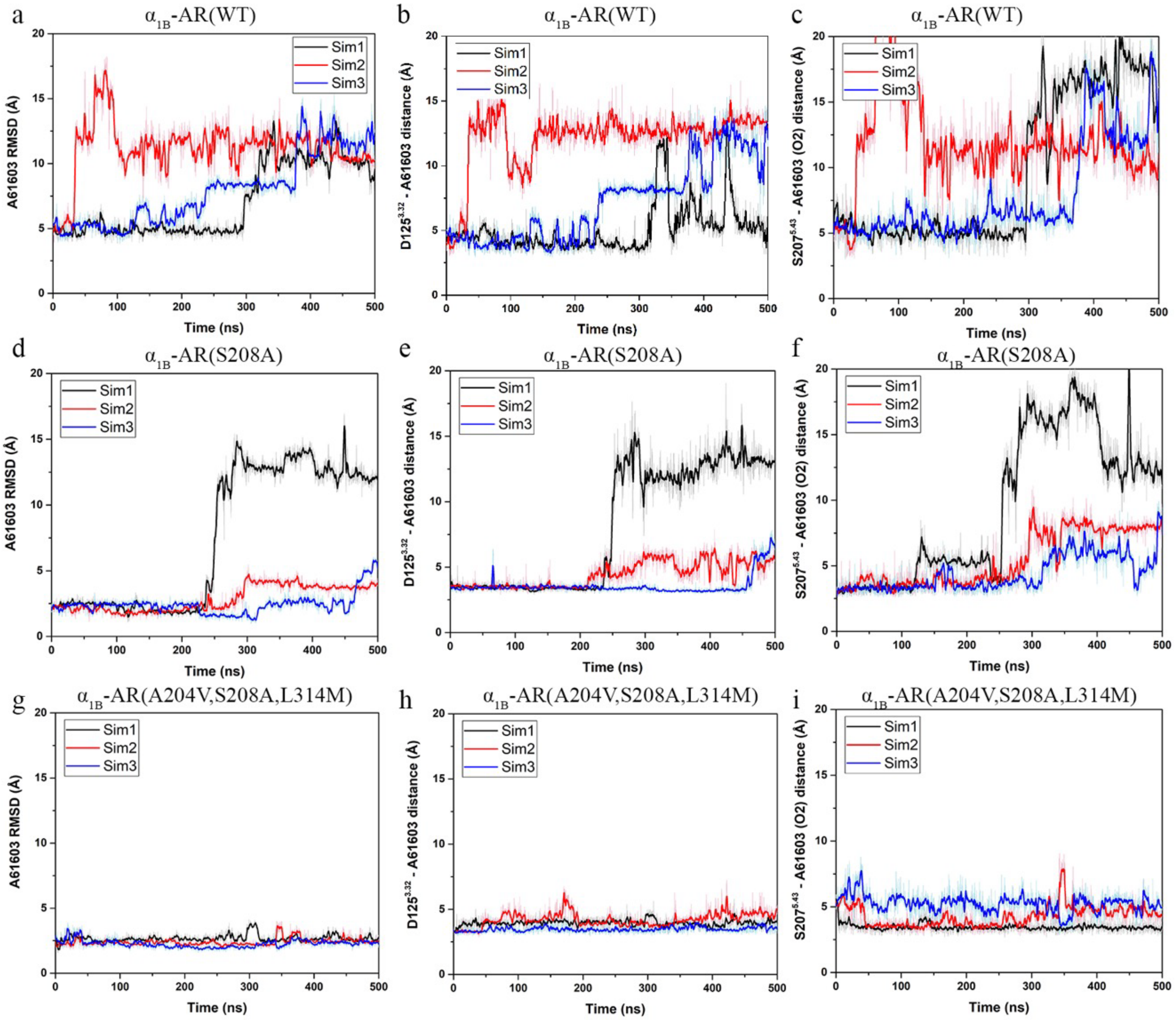

Supplementary Figure 10. **GaMD simulations of A61603 in complexes with wild-type and mutant α_{1B}-ARs.** (**a, d, g**) Time courses of A61603 RMSDs relative to the initial structures in the system of wild-type α_{1B}-AR (**a**), α_{1B}-AR(S208A) (**d**) and α_{1B}-AR(A204V,S208A,L314M) (**g**). (**b, e, h**) Time courses of the distance between the CG atom of D125$^{3.32}$ and N2 atom of A61603 in the system of wild-type α_{1B}-AR (**b**), α_{1B}-AR(S208A) (**e**) and α_{1B}-AR(A204V,S208A,L314M) (**h**). (**c, f, i**) Time courses of the distance between the OG atom of S207$^{5.43}$ and the O2 atom of A61603 in the wild-type of α_{1B}-AR (**c**), α_{1B}-AR(S208A) (**f**) and α_{1B}-AR (A204V,S208A,L314M) (**i**). Three independent 500 ns GaMD simulations are shown for each condition.

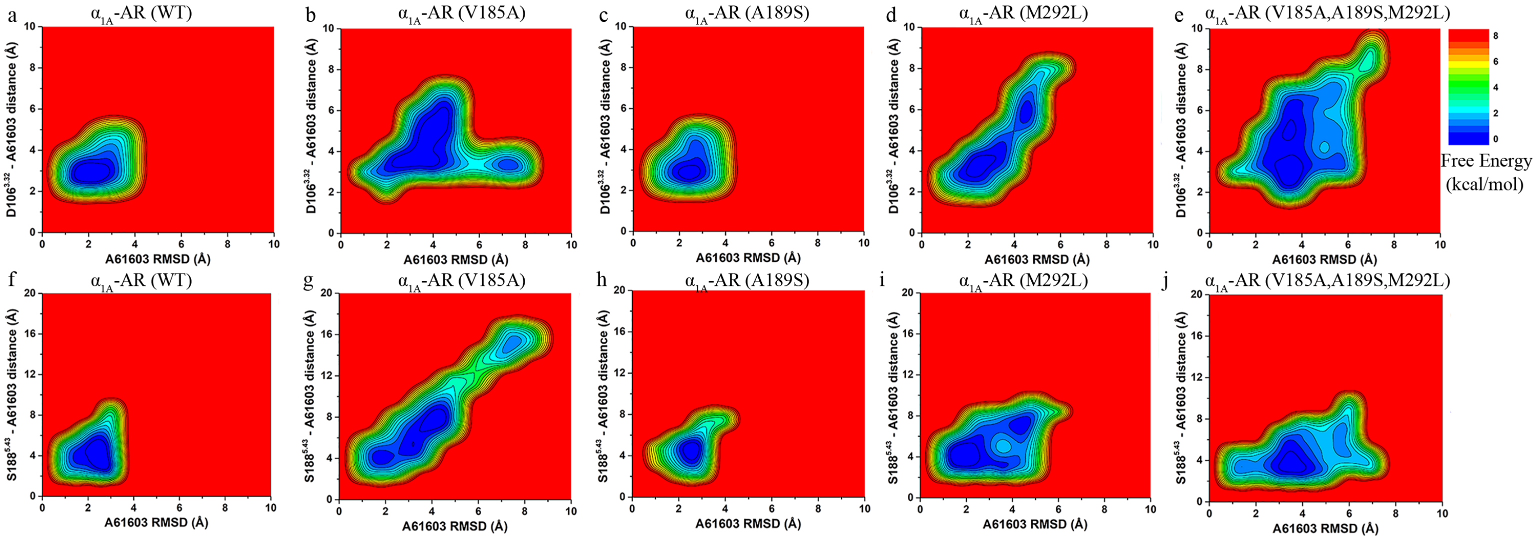

Supplementary Figure 11. **2D free energy profiles**.
(**a-e**) 2D free energy profiles of the A61603 RMSD relative to the distance between the CG atom of D106$^{3.32}$ and N2 atom of A61603 in the system of α_{1A}-AR (WT) (**a**), α_{1A}-AR (V185A) (**b**), α_{1A}-AR(A189S) (**c**), α_{1A}-AR(M292L) (**d**) and α1A-AR(V185A,A189S,M292L) (**e**). (**f-j**) 2D free energy profiles of the A61603 RMSD relative to the distance between the OG atom of S188$^{5.43}$ and O2 atom of A61603 in the system of α_{1A}-AR (WT) (**f**), α_{1A}-AR (V185A) (**g**), α_{1A}-AR(A189S) (**h**), α_{1A}-AR(M292L) (**i**) and α 1A-AR(V185A,A189S,M292L) (**j**).

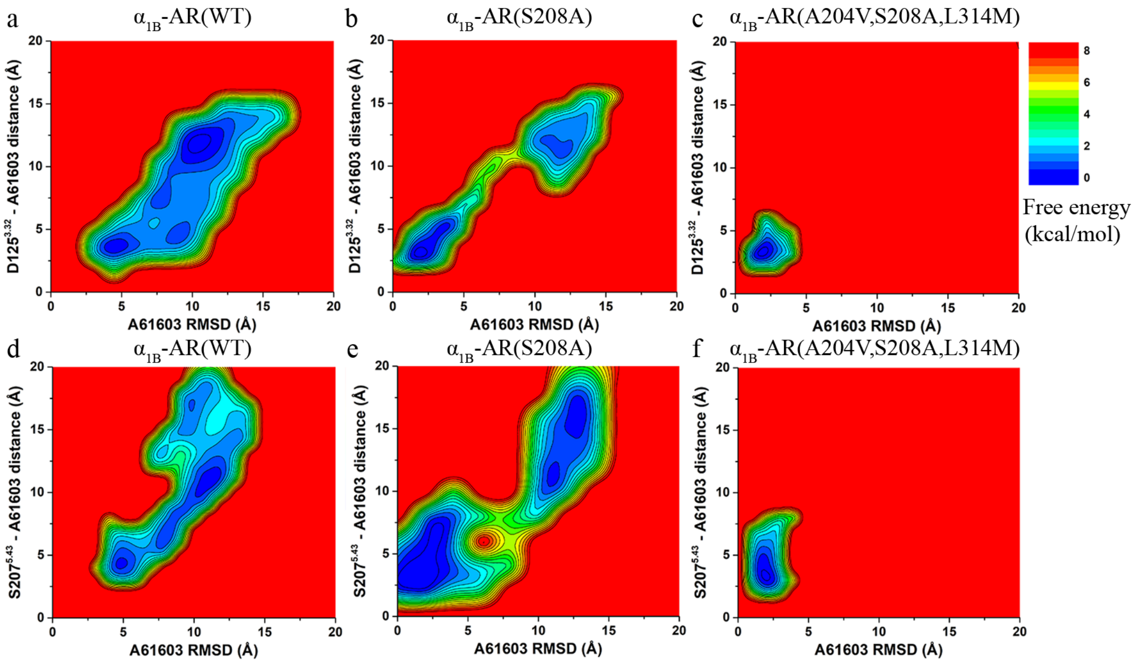

Supplementary Figure 12. **2D free energy profiles**.

(**a-c**) 2D free energy profiles of the A61603 RMSD relative to the distance between the CG atom of $D125^{3.32}$ and N2 atom of A61603 in the system of α_{1B}-AR (WT) (**a**), α_{1B}-AR (S208A) (**b**), and α_{1B}-AR(A204V,S208A,L314M) (**c**). (**d-f**) 2D free energy profiles of the A61603 RMSD relative to the distance between the OG atom of $S207^{5.43}$ and O2 atom of A61603 in the system of α_{1B}-AR (WT) (**d**), α_{1B}-AR (S208A) (**e**), and α_{1B}-AR(A204V,S208A,L314M) (**f**).

Comparison of A61603 and epinephrine conformations and orientations in the complexes with α_{1A}-AR and Gq

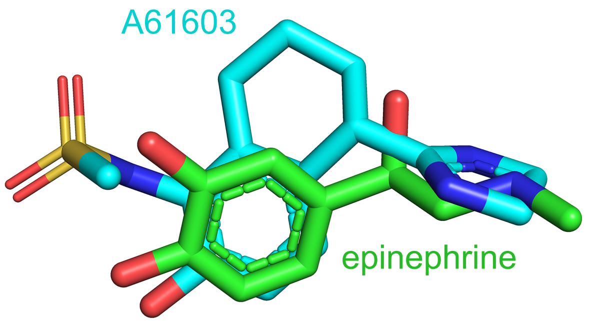

Supplementary Figure 13. Orientations of A61603 and epinephrine in the complex structures.
The conformations of A61603 and epinepherine in the cryo-EM structures are compared.

Supplementary Table 1. Cryo-EM data collection, refinement and validation statistics

	**A61603–α_{1A}-AR–Gq** (EMDB-41267) (PDB 8THK)	**Epinephrine–α_{1A}-AR–Gq** (EMDB-41268) (PDB 8THL)
**Data collection and processing**		
Magnification	81,000x	64,000x
Voltage (kV)	300	300
Electron exposure (e^-/Å^2)	50	52
Defocus range (μm)	-1.0 to -1.8	-1.0 to -1.8
Pixel size (Å)	1.07	1.076
Symmetry imposed	C1	C1
Initial particle images (no.)	1,497,830	880,531
Final particle images (no.)	360,489	219,834
Map resolution (Å) (Full/α_{1A}-AR–Gα focus)	2.62/2.86	3.07/3.04
FSC threshold	0.143	0.143
**Refinement**		
Initial model used (PDB/ AlphaFoldDB code)	6WHA and AF-P35348-F1	6WHA and AF-P35348-F1
Model resolution (Å)	2.9/2.4	3.4/3.0
FSC threshold	0.50 / 0.143	0.50 / 0.143
Map sharpening *B* factor (Å^2)	-50	-30
Model composition		
Non-hydrogen atoms	8,159	7,837
Protein residues	1,080	1,080
Ligands	1	1
B factors (Å^2)		
Protein	57.64	105.68
Ligand	48.76	123.23
R.m.s. deviations		
Bond lengths (Å)	0.002	0.003
Bond angles (°)	0.478	0.483
Validation		
MolProbity score	1.19	1.46
Clashscore	4.00	5.70
Poor rotamers (%)	0.95	0.79
Ramachandran plot		
Favored (%)	98.21	97.17
Allowed (%)	1.79	2.83
Disallowed (%)	0.00	0

Supplementary Table 2. Summary of the A61603 root-mean-square-fluctuation (RMSF) and binding free energy from MM/GBSA calculations on wild-type and mutant α_{1A}-ARs and α_{1B}-ARs. For each system, 1,000 frames were used for MM/GBSA calculations. Mean ± SD are shown.

α_{1A}-AR					
System	WT	V185A	A189S	M292L	V185A,A189S,M292L
A61603 RMSF (Å)	1.46 ± 0.10	2.36 ± 0.52	1.10 ± 0.09	1.74 ± 0.39	2.58 ± 0.33
A61603 binding free energy (kcal/mol)	-26.73 ± 0.22	-22.18 ± 0.73	-26.54 ± 1.89	-27.88 ± 1.53	-11.52 ± 2.47
α_{1B}-AR					
System	WT	S208A	A204V,S208A,L314M		
A61603 RMSF (Å)	4.28 ± 0.30	3.55 ± 1.97	0.98 ± 0.21		
A61603 binding free energy (kcal/mol)	-11.52 ± 2.47	-25.81 ± 2.67	-28.21 ± 2.80		
