# Supplementary material for: Structural basis of agonist specificity of α1A-adrenergic receptor
Source: Nat Commun. 2023 Aug 10;14:4819. doi: 10.1038/s41467-023-40524-2 (PMC10415349; doi:10.1038/s41467-023-40524-2)
Supplement: Supplementary file 3 — Reporting Summary [file 41467_2023_40524_MOESM3_ESM.pdf]

nature portfolio

Corresponding author(s): Xin-Yun Huang

Last updated by author(s): Jul 12, 2023

Reporting Summary

Nature Portfolio wishes to improve the reproducibility of the work that we publish. This form provides structure for consistency and transparency in reporting. For further information on Nature Portfolio policies, see our Editorial Policies and the Editorial Policy Checklist.

Statistics

For all statistical analyses, confirm that the following items are present in the figure legend, table legend, main text, or Methods section.

n/a	Confirmed	
☐	☒	The exact sample size (*n*) for each experimental group/condition, given as a discrete number and unit of measurement
☐	☒	A statement on whether measurements were taken from distinct samples or whether the same sample was measured repeatedly
☒	☐	The statistical test(s) used AND whether they are one- or two-sided<br>*Only common tests should be described solely by name; describe more complex techniques in the Methods section.*
☒	☐	A description of all covariates tested
☒	☐	A description of any assumptions or corrections, such as tests of normality and adjustment for multiple comparisons
☐	☒	A full description of the statistical parameters including central tendency (e.g. means) or other basic estimates (e.g. regression coefficient) AND variation (e.g. standard deviation) or associated estimates of uncertainty (e.g. confidence intervals)
☒	☐	For null hypothesis testing, the test statistic (e.g. *F*, *t*, *r*) with confidence intervals, effect sizes, degrees of freedom and *P* value noted<br>*Give P values as exact values whenever suitable.*
☒	☐	For Bayesian analysis, information on the choice of priors and Markov chain Monte Carlo settings
☒	☐	For hierarchical and complex designs, identification of the appropriate level for tests and full reporting of outcomes
☒	☐	Estimates of effect sizes (e.g. Cohen's *d*, Pearson's *r*), indicating how they were calculated

Our web collection on statistics for biologists contains articles on many of the points above.

Software and code

Policy information about availability of computer code

Data collection	Leginon System version beta
Data analysis	MotionCor2 1.2.1, Relion 4.0-beta, CTFFIND v4.1.8, CryoSparc v3.3.1, Coot v0.9.1, Phenix dev-4694, GraphPad Prism 9, ImageJ 1.52a, and NIS-Elements Advance Research 5.2.6.

For manuscripts utilizing custom algorithms or software that are central to the research but not yet described in published literature, software must be made available to editors and reviewers. We strongly encourage code deposition in a community repository (e.g. GitHub). See the Nature Portfolio guidelines for submitting code & software for further information.

Data

Policy information about availability of data

All manuscripts must include a data availability statement. This statement should provide the following information, where applicable:

- Accession codes, unique identifiers, or web links for publicly available datasets
- A description of any restrictions on data availability
- For clinical datasets or third party data, please ensure that the statement adheres to our policy

The cryo-EM reconstructions of the A61603–a1A-AR–Gq–scFv16 complex and the epinephrine–a1A-AR–Gq–scFv16 complex have been deposited in the Election Microscopy Data Bank (EMDB) under ID codes EMD-41267 and EMD-41268, respectively. The corresponding atomic models have been deposited in the Protein Data Bank (PDB) under ID codes 8THK and 8THL, respectively. Publicly available PDB entries used in this study are available under the accession codes 7B6W and 6WHA.

Human research participants

Policy information about studies involving human research participants and Sex and Gender in Research.

Reporting on sex and gender	N/A
Population characteristics	N/A
Recruitment	N/A
Ethics oversight	N/A

Note that full information on the approval of the study protocol must also be provided in the manuscript.

Field-specific reporting

Please select the one below that is the best fit for your research. If you are not sure, read the appropriate sections before making your selection.

[x] Life sciences [ ] Behavioural & social sciences [ ] Ecological, evolutionary & environmental sciences

For a reference copy of the document with all sections, see nature.com/documents/nr-reporting-summary-flat.pdf

Life sciences study design

All studies must disclose on these points even when the disclosure is negative.

Sample size	No Sample size calculations were performed. For cryo-EM samples, eight grids of each sample were pre-screened to identify the optimal grid for data collection. The number of grids screened were random and was not limited by any experimental parameter. The sample size was deemed sufficient as it allowed to determine the structures.
Data exclusions	No data were excluded from the analyses.
Replication	The Ca2+ assays were repeated three times, and the data are represented as mean ± SD of the three independent experiments. All attempts at replication were successful.
Randomization	Division of particles into random halves is automatically performed during 3D reconstruction by Relion 4.0-beta and CryoSparc v3.3.1.
Blinding	Blinding is not applicable for this study, as group allocation is not used.

Reporting for specific materials, systems and methods

We require information from authors about some types of materials, experimental systems and methods used in many studies. Here, indicate whether each material, system or method listed is relevant to your study. If you are not sure if a list item applies to your research, read the appropriate section before selecting a response.

Materials & experimental systems

n/a	Involved in the study	
[x]	[ ]	Antibodies
[ ]	[x]	Eukaryotic cell lines
[x]	[ ]	Palaeontology and archaeology
[x]	[ ]	Animals and other organisms
[x]	[ ]	Clinical data
[x]	[ ]	Dual use research of concern

Methods

n/a	Involved in the study	
[x]	[ ]	ChIP-seq
[x]	[ ]	Flow cytometry
[x]	[ ]	MRI-based neuroimaging

Eukaryotic cell lines

Policy information about cell lines and Sex and Gender in Research

Cell line source(s)	Insect cell line Sf9 was obtained from Expression Systems. HEK293T cell lines were obtained from ATCC.
Authentication	Authentication was not performed for this study.
Mycoplasma contamination	All cell lines were tested negative for mycoplasma contamination.

Commonly misidentified lines<br>(See ICLAC register)	No commonly misidentified cell lines were used.
